# Molecular Dissection of the Antibody Response: Opportunities and Needs for Application in Cattle

**DOI:** 10.3389/fimmu.2020.01175

**Published:** 2020-06-12

**Authors:** Ruben Barroso, W. Ivan Morrison, Liam J. Morrison

**Affiliations:** Roslin Institute, Royal (Dick) School of Veterinary Studies, University of Edinburgh, Midlothian, United Kingdom

**Keywords:** B cell, immunoglobulin, bovine, single cell analysis, ultralong HCDR3 domain

## Abstract

Improving understanding of the bovine adaptive immune response would equip researchers to more efficiently design interventions against pathogens that impact upon food security and animal welfare. There are features of the bovine antibody response that differ substantially from other mammalian species, including the best understood models in the human and mouse. These include the ability to generate a functionally diverse immunoglobulin response despite having a fraction of the germline gene diversity that underpins this process in humans and mice, and the unique structure of a subset of immunoglobulins with “ultralong” HCDR3 domains, which are of significant interest with respect to potential therapeutics, including against human pathogens. However, a more detailed understanding of the B cell response and the production of an effective antibody response in the bovine is currently hampered by the lack of reagents for the B cell lineage. In this article we outline the current state of knowledge and capabilities with regard to B cell and antibody responses in cattle, highlight resource gaps, and summarize recent advances that have the potential to fundamentally advance our understanding of this process in the bovine host.

## Introduction

The molecular basis of how antibody repertoires are generated is broadly similar between mammalian species. Rearrangement of genes encoding immunoglobulin heavy and light chains during B cell development, from a pre-existing library of variable gene segments, results in each B cell expressing a unique immunoglobulin specificity. Immunoglobulin diversity is further refined by somatic mutation of the immunoglobulin genes during development of an immune response, which enables selection of B cells expressing antibodies with enhanced affinity for the immunogen. Studies of immunoglobulins in domestic animal species have highlighted certain unique features. One such example is that Camelids produce a subset of immunoglobulins composed only of a heavy chain, in which antigen recognition involves only one variable region. This has allowed isolation of these single heavy chains and their expression as recombinant antibodies, referred to as nanobodies, for various practical applications (1). Compared to humans and mice, cattle and sheep have a more restricted repertoire of immunoglobulin variable gene segments, but they compensate for this by utilizing antigen-independent somatic mutation of their rearranged immunoglobulin genes to generate further sequence diversity. Another distinct feature in cattle is that ~10–15% of immunoglobulins possess an ultralong heavy chain CDR3 domain. In contrast to conventional antibodies where antigen recognition involves interaction with six hypervariable loops (or complementarity determining regions–CDRs)–three on the heavy chain and three on the light chain—recognition of antigen by these ultralong antibodies is determined predominantly by the HCDR3, which has an extended stalk-knob like structure (2–5).

The capacity to analyse antibody responses against infectious agents at the single B cell level provides a powerful means to identify the biological properties of individual antibody specificities, including their potential role in immunity. Until recently, such analyses have proved difficult in outbred species. Techniques developed in the 1970s for generating monoclonal antibodies in mice and rats were not readily applicable to other species, because of the absence of suitable myeloma cell lines for use as fusion partners. Attempts to use murine myeloma cell lines with bovine B cells to generate heterohybridomas had some success in producing bovine monoclonal antibodies, but these systems were not sufficiently efficient to allow their routine use (6–8). A further factor that constrained the ability to analyse antibody responses at the clonal level was the limited capacity of antibody-producing cells to proliferate, as they undergo terminal differentiation as plasma cells. This is in marked contrast to antigen-specific T cells, which can be propagated and cloned *in vitro*, allowing analyses of their specificity at the clonal level. In the last few years, advances in the sensitivity of methods to examine gene expression at the single cell level have opened up new opportunities to analyse B cell responses, including the isolation of expressed immunoglobulin genes from individual B cells.

This paper aims to provide a brief review of new and emerging approaches to interrogating bovine antibody responses, focusing particularly on analyses of responses at the single B cell level.

## Advances in Clonal Analyses of Antibody Responses in Outbred Species

In the last few years, methods have been established for generating antigen-specific human monoclonal antibodies from B cells isolated *ex vivo* from humans mounting an antibody response. These methods are based on the ability to enrich for specifically reactive B cells and the capacity to isolate and express immunoglobulin genes from single responding B cells. Enrichment for antigen-specific B cells has relied either on use of fluorescently labeled antigen tetramers to identify and isolate antigen-specific B cells or isolation of plasmablasts and plasma cells using surface markers expressed specifically on these activated B cell populations. Rapid methods for isolation and expression of immunoglobulin heavy and light chain genes from single B cells have allowed analyses of the antibody specificities. Such approaches have proved to be highly successful in generating novel data on the fine specificity of human antibody responses to a number of pathogens, most notably influenza and Ebola viruses (9, 10).

The ability to conduct similar analyses of antibody responses in cattle would represent a major advance, particularly with respect to identification of antibody targets for use in vaccination. Many pathogens induce antibody responses to multiple antigens, only some of which play an important role in immune protection. The capacity to screen the biological activities of monoclonal antibodies induced in the target species, provides a direct means of identifying antigens that are likely to be immunogenic. In some diseases, immune responses are dominated by antibodies against antigens that vary between pathogen strains, leading to strain-specific immunity (e.g., foot and mouth disease virus). In such cases, interrogation of the fine specificity of the response at the clonal level, offers the means of identifying subdominant cross-reactive antigenic specificities with potential for vaccination.

## Reagents for Studying B Cell Responses in Cattle

The ability to apply these new technologies to studies of bovine B cell responses has been constrained by a paucity of reagents for studying B cell differentiation. Studies of human B cell responses are able to utilize a suite of reagents developed against surface markers, which enables relatively precise characterization and placement of B cells within the differentiation cascade. Identification of particular stages of differentiation frequently relies on the use of combinations of several markers, and in some instances consideration of their levels of expression.

Two distinct lineages of B cells, B-1 and B-2, have been identified in humans and mice. In contrast to conventional B-2 cells, which cooperate with helper T cells and undergo Ig isotype switching and affinity maturation within germinal centers, B-1 B cells have minimal requirement for auxiliary signals and respond rapidly by producing predominantly IgM (11, 12). The majority of B-1 cells are CD5^+^ (referred to as the B-1a subset), with a minor subset being CD5^−^ (B-1b subset). In cattle, expression of surface CD5 has been used as a marker for B-1a B cells, which represent ~20–25% of B cells in PBMC (13). CD5^+^ B cells play a prominent role in bovine immune responses to a number of pathogens, including *Trypanosoma congolense* (14), foot and mouth disease virus (15) and Bovine Leukosis Virus (16). In the case of *T. congolense*, the percentage of CD5^+^ B cells in peripheral blood approximately doubles during the first 3–4 weeks of infection.

Of greater relevance to the present discussion are B-2 B cells, which undergo a complex series of differentiation events during generation of an antibody response. Interaction of mature-naïve B cells with antigen via the B cell receptor (BCR), which is associated with a complex of proteins (CD19, CD21 and CD79) that are responsible for co-stimulation, promotes B cell activation and differentiation (17, 18). Multiple changes in cell surface phenotype occur once the B cells have been activated, including increased levels of CD40, CD69, CD80 and CD86. Up-regulation of CD40, coupled with antigen uptake by specific B cells, enables them to interact with antigen-specific T cells in the follicles of secondary lymphoid organs, leading to germinal center formation and further B cell differentiation, including Ig isotype switching and affinity maturation. The latter involves a clonal selection process, in which antigen-specific B cells with the highest affinity are selected for survival and clonal expansion. Finally, B cells either differentiate into long-lived memory cells or develop to plasmablasts and antibody-secreting plasma cells. Increased expression of surface CD27 is an important marker for memory cells, although they show considerable heterogeneity in phenotype and function (19, 20). Among the phenotypic changes that occur during differentiation to plasma cells is increased levels of expression of CD38, which is frequently exploited for identifying antibody-secreting cells (21).

In contrast to human B cells, there is a distinct lack of antibody reagents that enable discrimination between the different states of differentiation of B-2 B cells in cattle. Apart from surface immunoglobulin and CD21, there are no well-defined pan-B cell markers in cattle. Although IgD is used as one of the surface markers of naive human B cells, its existence in cattle was only demonstrated in 2006 (22) and there is only one report of expression of the protein on a minor subset of bovine B cells (23). There are also no monoclonal antibody reagents that can be used to identify plasmablasts and plasma cells. Similarly, memory B cell markers are not well-developed for cattle. Therefore, the ability to resolve and understand the intricacies of the bovine B cell response is substantially hampered at present, and requires investment to generate the tools required to fill this gap; this is starting to be addressed by initiatives such as the Veterinary Immunological Toolbox (https://www.immunologicaltoolbox.co.uk/).

## Genomic Organization of Immunoglobulin Genes in the Cow and Diversity Generation

It is now known that bovine B cells express five isotypes of immunoglobulin: IgM, IgD, IgG, IgE and IgA, with the IgG isotype differentiated into three sub-isotypes (IgG1, IgG2 and IgG3), and IgM into two sub-isotypes (24, 25). Until relatively recently, annotation of the bovine heavy and light chain genomic loci was incomplete. Of the genes that encode the immunoglobulin antigen binding domains [heavy and light chain variable (V), diversity (D) and joining (J) segments], which are generated by VDJ recombination, cattle differ substantially from humans and mice, in particular with respect to the comparative paucity of variable gene content. Cattle have only twelve genes encoding functional heavy chain variable gene segments (IGHV—located on chromosome 21), and all belong to one subgroup, IGHV1 (compared to seven diverse subgroups in humans), with a number of pseudogenes also described in both IGHV1 and two further subgroups, IGHV2 and IGHV3. Only four of the twelve documented heavy chain joining gene segments (IGHJ) and sixteen of the twenty-three diversity gene segments (IGHD) found in cattle appear to be functional (25). Additionally, compared with humans and mice, available data suggest that cattle have a more restricted set of putatively functional light chain genes (26). Most vertebrates express two light chain isotypes: kappa (κ) and lambda (λ). However, the bovine light chain repertoire is dominated by the expression of λ genes [κ usage represents ~5% of the expressed antibody repertoire (27)], and predominantly by one subfamily, V_λ_1. V_λ_ genes are clustered close to the J_λ_ and C_λ_ cluster on chromosome 17 (28) and V_κ_ genes on chromosome 11 (26). In cattle, the limited data available suggest the light chain may have a subsidiary role in antigen recognition, with most antigen binding being driven by the heavy chain variable region. Recent x-ray crystallography data on the structure of two bovine IgG antibodies support this assertion by showing that the heavy chain predominantly contributes to the antigen-combining site (29, 30). When the light chains were exchanged between these two antibodies, antigen recognition by one of the antibodies (but not the other) was substantially reduced and structurally this was associated with a subtle change in the orientation of the associated heavy chain. An earlier study of a poly-specific IgM long-CDR3 antibody had also demonstrated a predominant role of the heavy chain in antigen recognition, although interaction with some antigenic ligands was influenced by the light chain (29).

The information on the genomic architecture of the bovine immunoglobulin loci has been derived from work on European *Bos taurus* breeds, with the most complete genome assembly and associated resources deriving from a Hereford cow (31–34). Immune gene loci tend to be highly repetitive by nature, and therefore difficult to accurately assemble without the use of resource-intensive sequencing technologies that enable accurate construction across large stretches of multiple and similar gene members—for example, long-read or chromatin-linking sequencing approaches. While such genomic resources are being developed for other breeds [e.g., Brahman *Bos indicus* (35)], there are still too few genomes sequenced to a sufficient depth across diverse cattle breeds and lineages to enable assessment of the degree of immunoglobulin locus polymorphism, and how that may impact upon antibody expression and function. This is the focus of increasing effort (e.g., the Bovine Pan Genome Consortium), and increasing the genomic resources across breeds and lineages will be important in functionally linking genomic diversity to phenotypic diversity with respect to the bovine antibody response.

The limited repertoire of germline variable gene segments in cattle has been proposed to be offset by the occurrence of somatic hypermutation in rearranged B cells prior to exposure to antigen, thus generating greater diversity and expanding the B cell repertoire (36, 37). There is evidence from studies in both sheep and cattle (36, 38) that the ileal Peyer's patch is a major site of this antigen-independent somatic mutation. This organ, which differs histologically from conventional Peyer's patches, develops with the kinetics of a primary lymphoid organ (i.e., similar to the thymus). In sheep, the ileal Peyer's patch undergoes significant development during the latter half of gestation, with further enlargement in the first few months of life, and gradual involution from about 3 months onwards (39).

Bovine HCDR3 length on average is longer than in other vertebrates such as humans or mice (bovine HCDR3 ranging from <10 to at least 67 amino acids in length, in contrast to 4–36 amino acids in humans) (2, 5, 22, 24, 40, 41). It has been known for many years that a proportion (~10%) of bovine immunoglobulin transcripts contain unusually long HCDR3 domains up to and beyond 60 amino acids long (40, 42–45)—often termed “ultralong” HCDR3 domains. Resolution of the structure of these antibodies identified an unusual and relatively conserved stalk-knob protrusion, which comprised the HCDR3 antigen-binding domain (2, 3, 5). Formation of the stalk structure is facilitated by the presence of several disulphide bonds. The ultralong antibodies described thus far all utilize a single variable gene (IGHV1-7) and diversity gene (IGHD8-2) donor (2, 41), and the few paired heavy and light chain data available also suggest utilization of a limited number of λ light chain V genes (46). Based on analyses of the sequences of multiple long HCDR3 antibodies, a recent study by Deiss et al. (41) has identified a number of key features of the rearranged genes encoding these antibodies. Firstly, they confirmed the almost exclusive use of the IGHV1-7 gene segment and showed that this variable gene contains an internal 8-nucleotide duplication (which contributes to formation of the elongated stalk structure). They also found that, in contrast to other IGHV gene rearrangements, the IGHV1-7 CDR1 and CDR2 regions contain a low frequency of mutations, whereas the CDR3 regions of the same genes show very high levels of mutation compared to the germline sequence (41). This relative conservation of CDR1 and CDR2 sequences is consistent with evidence that these regions have little involvement in antigen binding but rather play a structural role in the long HCDR3 antibodies, whereas the knob-like structure formed by the CDR3 region is the primary antigen-binding site. Direct evidence for the latter was provided by the demonstration that removal of the “knob” sequence ablated antigen binding by the modified antibody (2). Deiss et al. also identified an unusually high degree of deletion events in the HCDR3 domains of long antibodies (predominantly in the IGHD8-2 segment), including deletions that alter the reading frame, thus contributing to ediversity in both the length and sequences of the CDR3 segments and hence structural diversity of these antibodies (41). They hypothesized that this may also be mediated by the enzymatic driver of somatic hypermutation, activation induced cytosine deaminase (AID). This mechanism has been proposed to be a means of generating structural diversification through modification of the pattern of disulfide bond formation (41). This is facilitated by an unusual codon bias in HCDR3, which predicates mutation to cysteine (particularly in the IGHD8-2 segment codons) during bovine VDJ recombination, resulting in the generation of diversity in structure due to the making and breaking of di-sulfide bonds between paired/unpaired cysteines (2, 47). This diversification mechanism has also been shown to operate in conventional length bovine antibodies (47). Analyses of the sequences of re-arranged bovine Ig genes has additionally indicated evidence of a low frequency of gene conversion events in both light and heavy chains that involves short nucleotide segments from light and heavy chain pseudogenes (48, 49), potentially providing a further means of generating sequence diversity–although current data are limited and its importance has yet to be fully determined. In summary, the long HCDR3 antibodies exemplify the bovine host's adaptations to generating antibody diversity from a limited germline repertoire–the combination of codon bias and (possible AID-mediated) targeted deletions resulting in changing of the pattern of cysteine pairs, generating a remarkable ability to create structural diversity in epitope-binding domains, despite being restricted to the use of a single V and D segment.

While the function of these ultralong antibodies remains unclear [interestingly the proportion of ultralong antibodies is significantly higher in neonatal calves (50)], their unusual structure quickly raised the hypothesis that such antibodies could bind to antigen epitopes that were not accessible to conventionally structured immunoglobulins. For example, sites on bacterial pore proteins or proteins embedded within the complex surface coat of parasitic pathogens, which are hidden from conventional antibodies, may be potential targets. There has also been significant interest in application of ultralong antibodies to non-bovine pathogens and their exploitation for development as potential therapeutics, in particular for human pathogens such as HIV (46)—the potential for therapeutics of relevance to veterinary pathogens is also clearly a possibility that is currently underexploited. However, the exact roles that these antibodies play during natural immune responses in cattle, or, for example, whether they may be an important factor in the efficacy of immune responses induced by vaccines, are unclear. Additionally, all studies analyzing long HCDR3 antibodies have examined European *Bos taurus* cattle—although the long HCDR3 antibody expression levels been shown to be consistent across several European *B. taurus* breeds, current data on, for example, long HCDR3 antibody expression data in *Bos indicus* or African *B. taurus* breeds, and any role they may play across the very different infectious disease contexts that such breeds are exposed to, are all currently unknown. These are all areas that clearly merit further research.

## *In vitro* Culture of Activated B Cells

Although most studies of antibody responses at the single cell level have focused on analyses of actively responding B cells harvested *ex vivo*, in recent years there have been attempts to analyse the antibody repertoire of memory B cells (51). Since memory B cells are normally present at low frequencies, this approach is dependent on use of culture systems to activate and expand the memory B cells. This in turn requires precise phenotyping reagents (52). A number of studies have reported successful establishment of *in vitro* culture systems that allow expansion of human memory B cell populations and differentiation to immunoglobulin secretion (53). These studies have used combinations of factors that stimulate activation, proliferation, and differentiation, coupled with inhibition of apoptosis, of B cells to maintain growth *in vitro*, albeit for a limited period of time.

Systems for culturing B cells have attempted to mimic the events that drive B cell development during antibody responses *in vivo*. The stimulation of B cells by crosslinking of the BCR with anti-IgM antibodies is well-established as a means of mimicking antigen stimulation (54). Uptake and processing of antigen by specific B cells enables them to present the antigen to T cells, which provide co-stimulatory signals by interaction of CD40 on the activated B cells with CD40 ligand (CD40L) on the T cells. This process can be mimicked *in vitro* by stimulation of B cells with soluble CD40L (55, 56). Activation *in vitro* via CD40 promotes an increase of levels of IL-21 receptor on the B cell surface (57). Among the cytokines that also contribute to B cell activation, IL-21, induced by T cells upon interaction with B cells is a key stimulus for B cell proliferation and differentiation (58). One of the main surface ligands involved in B cell survival is the BAFF receptor (BAFFR) that binds BAFF (B cell activating factor of the TNF family). Other similar related receptors that bind BAFF, are TACI and BCMA, which can also bind APRIL (α proliferation-inducing ligand), and these play a key role in preventing cell death and increasing plasma cell survival (59).

This knowledge has been applied to successfully culture activated porcine B cells, taking advantage of the cross-reactivity of the human reagents with porcine B cells (60). Addition of IL-21 plus CD40L to purified pig B cells resulted in activation and proliferation over a 4-day period, and inclusion of BAFF and APRIL maintained the viability of the cells for 7 days. Secretion of low levels of both IgM and IgG by these cultures was detected on day 7 of culture indicating differentiation of some of the activated B cells. The reagents used in this study also cross-react with bovine B cells and we have been able to obtain similar activation, proliferation and maintenance of bovine B cells similar to that reported by Rahe and Murtaugh (60). The development of phenotyping reagents that allow identification of bovine memory B cells will enable these culture systems to be used to amplify memory cell populations prior to clonal analyses of their specificities.

## Isolation of Antigen-Specific B Cells

The isolation of antigen-specific B cells is a critical step in the ability to evaluate and analyze the bovine humoral immune response, particularly responses to either specific pathogens or vaccination. In addition, due to the unique properties of bovine antibodies as described above, the isolation of antigen-specific cells is a route to explore their potential relevance as novel molecular tools for research or therapeutic use.

**Figure 1:**
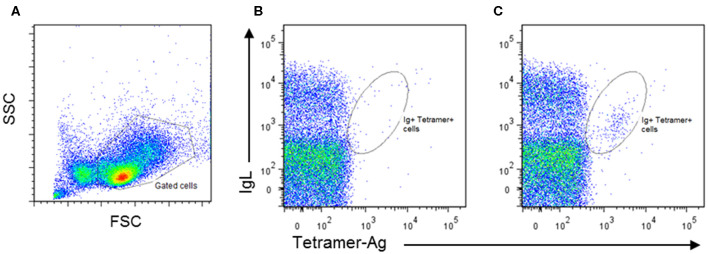
Identification of bovine antigen-specific B cells in peripheral blood mononuclear cells (PBMCs) of an antigen-immunized calf by staining with Phycoerythrin-labeled antigen tetramers: **(A)** PBMCs isolated from naïve and immunized calves were stained by two-color immunofluorescence with a monoclonal antibody (IL-A58) specific for bovine immunoglobulin light chain (IgL) and antigen tetramer. Representative plots of stained cells from a naïve animal **(B)** and an immunized animal **(C)** are presented, showing the presence of an IgL^+^ Tetramer-Ag^+^ population in the immunized animal. Approximately 1% of IgL^+^Tetramer-Ag^+^ B cells were detected in the immunized calve, compared to <0.2% in the unimmunized control.

Populations of human B cells enriched for antibody-producing cells have been isolated from blood by flow cytometry using a combination of cell surface markers, including CD19, CD20, CD38, and CD71 (61). In the absence of such markers for cattle, the ability of antibody-producing cells to bind fluorescently labeled tetramerised antigen offers an alternative. This approach is challenging because of the low frequencies of antigen-producing B cells in peripheral blood, and the short time window during which these cells are present at sufficient frequency for detection by flow cytometry. Moreover, antigen tetramers do not detect all antibody-producing cells, as mature plasma cells lose expression of surface Ig upon transition from plasmablasts (62). We have employed established methods (63) to produce streptavidin-labeled tetramers incorporating a recombinant protein from the major cattle pathogen, *Trypanosoma congolense* and used these tetramers to monitor the blood of calves immunized with this antigen. These experiments revealed the presence of a small population of surface Ig^+^ tetramer^+^ cells, detectable for several days after the third dose of antigen administered in adjuvant (Figure 1). In Giemsa–stained cytospin preparations the positive cells exhibited a plasmablast morphology. Further studies are underway to isolate and analyse the immunoglobulin genes expressed by these B cells.

## Immortalisation of B Cells by Infection With *Theileria annulata*

**Figure 2:**
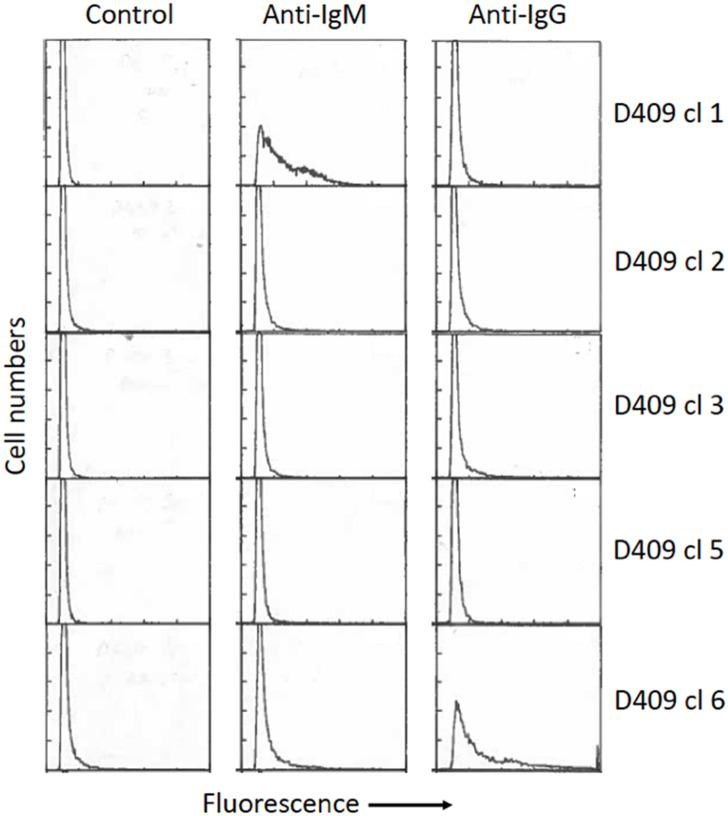
Surface phenotype of five cloned B cell lines infected with *Theileria parva*, as described by Baldwin et al. (65). Briefly, surface Ig+ B cells (>98% purity) were isolated by cell sorting from healthy resting peripheral blood mononuclear cells and infected *in vitro* by incubation with *T. parva* sporozoites, followed by cloning at limiting dilution in 96-well round-bottom plates. The cells were phenotyped 8 weeks after infection by staining with monoclonal antibodies specific for bovine IgM and IgG (Mab B5/4 and IL-A2, respectively) followed by fluorescein-labeled anti-mouse Ig. Significant levels of IgM or IgG expression were detected only on two of the clones (clone 1–IgM; clone 6–IgG). The percentages of positive gated cells for each clone are (Clone 1: Control−1%, IgM−54%, IgG−6%. Clone 2: Control−3%, IgM−6%, IgG−6%. Clone 3: Control−2%, IgM−3%, IgG−10%. Clone 5: Control−2, IgM−4%, IgG−4%. Clone 6: Control 5%, IgM−2%, IgG−47%). Controls were incubated with secondary antibody only. All clones were negative for T cell markers (CD2, CD4, CD8)–data not shown.

One of the potential uses of isolated antigen-specific B cells is to transform the cells to allow clonal expansion of the populations and potentially examine antibody secretion. Some species of *Theileria* parasites are able to infect and transform bovine lymphocytes. *Theileria* are tick-borne apicomplexan protozoa found in tropical and subtropical regions of the world. The most important species in cattle are *Theileria annulata* and *Theileria parva* (64). Both species infect leukocytes: *T. parva* infects T and B lymphocytes, while *T. annulata* infects monocytes and B cells (65, 66). A characteristic feature of infection with both parasites is that they induce activation and proliferation of the cells they infect (67), during which the parasites divide synchronously with the host cells (68). This relationship, coupled with inhibition of apoptosis of the host cells by the parasite (69) results in clonal expansion of the cells initially infected by the parasite. These properties enable the infected cells to be maintained as continuously growing cell lines *in vitro*, and such cell lines can be initiated by *in vitro* infection of leukocytes with the tick-derived infective stage of the parasite, the sporozoite. In previous studies, we examined the phenotype of purified resting Ig^+^ B cells several weeks after infection *in vitro* with *T. parva*. Most infected cells were found to gradually lose surface expression of immunoglobulin, although analyses of cloned populations revealed continued Ig expression, either IgM or IgG, by some clones (70) (Figure 2). Similar gradual loss of Ig expression has also been observed in B cells infected with *T. annulata*. However, expression of Ig by B cells in the early stages after infection by *Theileria* was not studied, nor was the susceptibility of activated B cells to infection examined in these experiments. In recent studies, we have shown that purified tetramer^+^ B cells are similarly susceptible to infection with *T. annulata*. However, similar to previous findings with infected resting B cells, following cloning and expansion of the cloned populations over a 3–4 week period, only a subset of the clones secreted antibody.

These preliminary finding suggest that this system could be used to obtain cells secreting antibody with particular antigenic specificities, but may not be suitable for direct large-scale clonal analyses of antibody responses at the level of Ig secretion. Nevertheless, the generation of cloned transformed B cells from antigen-specific B cells could prove to be a valuable resource of immortalized cells from which the rearranged Ig heavy and light chain pairs can be retrieved for further analyses. However, further studies are required to explore the full potential of this system.

## Immunoglobulin Genes Expressed by Single B Cells

While advances have been made in our understanding of the genomic repertoire of bovine immunoglobulin loci, we still have only limited data on the usage of these gene families in generating functional, effective antibody proteins. This partly stems from the difficulty in deconvoluting data generated from cell pools or populations into that relevant at the single cell level—this is in most cases an insurmountable bioinformatic challenge, whether short or long read sequencing approaches are used. An obvious route to gaining data at this level is to analyse gene expression of multiple single cells, rather than averaging gene expression across RNA extracted from populations. There are several factors required to do this—one being an ability (within the context of a response to a particular immunogen) to identify and isolate multiple single antigen specific cells (current limitations and challenges around this are outlined above). Despite this challenge, data on single cells, yielding paired heavy and light chain sequences, are emerging in bovine studies, although still only from small numbers of cells.

The ability to analyse single cell data at scale from humans has significantly advanced in recent years. Single cells can be isolated by various routes [micro-dissection, flow cytometry, microfluidics and droplet-based methods (71)], each of which have their advantages and limitations. Droplet-based methods in particular have led to a step change in terms of scale, providing the ability to potentially analyse thousands of single cells (72–74). The challenge of analyzing the VH and VL sequences of B cells has been to some extent overcome in human studies by isolating single cells within emulsion droplets, in which the cells are lysed and mRNA captured by poly-dT beads. From this substrate physically linked VH and VL transcripts are generated through overlap-extension reverse-transcription PCR (OE RT-PCR), effectively splicing the VH and VL amplicons together, which can then be resolved into paired VH and VL chain data (75). This has resulted in novel insights in terms of VL and VH pairing and use, and the identification of broadly virus neutralizing antibodies (76). However, while providing a substantial improvement on individual VH and VL PCRs from isolated cells, the OE RT-PCR approach is still technically indirect, with the full-length variable sequence inferred from assembly of several partial sequences because of the limitations of sequencing technologies. A recent development, termed sc-BCRseq, applied barcodes to fragmented VH and VL sequences from single B cells within droplets, importantly then providing confident downstream assembly into full length paired VH and VL sequences (77). This approach also can be employed in a high throughput manner, and was successfully applied to 250,000 B cells and enabled high resolution analysis of antibody lineages in response to immunization (77). All of these approaches still have their challenges, one particular issue with B cells (if an aim is to analyse antibody response development through the B cell lineage) being sensitivity bias—the increased Ig transcript levels in plasmablasts meaning they are over-represented in expression data.

While the advances in single cell technologies present exciting future possibilities when applied to the bovine antibody response, in the context of analyzing antibody responses to a specific antigen, the initial step in identifying and isolating antigen-specific B cells is still necessary. Thus, the generation of B cell reagents that allow more precise analysis of single cells remains a priority in order to fully realize our ability to analyse the antibody response in cattle.

## Summary and Conclusions

Our understanding of the bovine B cell and antibody response has advanced significantly in recent years, with genomic and experimental data resolving the unique manner in which the cow generates immunoglobulin diversity from a restricted germline VH repertoire. This has included the characterization of ultralong HCDR3 domain antibodies and their structure, and consequent interest in their potential application to novel therapeutics. However, our ability to advance understanding of many aspects of the antibody response is restricted by a lack of reagents for bovine B cells, in particular those that allow identification and characterization of B cells at different stages of differentiation along the B cell lineage. Such tools would enable more detailed analysis of the initiation, progression and maturation of an effective antibody response, as well as the ability to address specific questions such as the role of HCDR3 antibodies during infection or in response to vaccination. The ability to isolate antigen-specific B cells is also key to facilitating analyses within the context of infection or vaccination, and we have outlined potential routes to how this could be achieved–the development of better reagents for bovine plasma cells/plasmablasts would certainly significantly enhance this capability. Finally, application of single cell sequencing technologies has the potential to revolutionize the analysis of B cell responses, enabling the isolation of paired heavy and light chain data from hundreds of thousands of B cells. Therefore, with investment in the development of key reagents combined with single cell sequencing at scale, we are poised to enter an era that can transform our understanding of the bovine antibody response.

## Author Contributions

RB, LM and WM wrote the manuscript.

## Conflict of Interest

The authors declare that the research was conducted in the absence of any commercial or financial relationships that could be construed as a potential conflict of interest.
